# Grassmann Extrapolation of Density Matrices for Born–Oppenheimer Molecular Dynamics

**DOI:** 10.1021/acs.jctc.1c00751

**Published:** 2021-10-08

**Authors:** Étienne Polack, Geneviève Dusson, Benjamin Stamm, Filippo Lipparini

**Affiliations:** † Laboratoire de Mathématiques de Besançon, UMR CNRS 6623, Université Bourgogne Franche-Comté, 16 Route de Gray, 25030 Besançon, France; ‡ Department of Mathematics, RWTH Aachen University, Schinkelstr. 2, 52062 Aachen, Germany; § Dipartimento di Chimica e Chimica Industriale, Univeristà di Pisa, Via G. Moruzzi 13, I-56124 Pisa, Italy

## Abstract

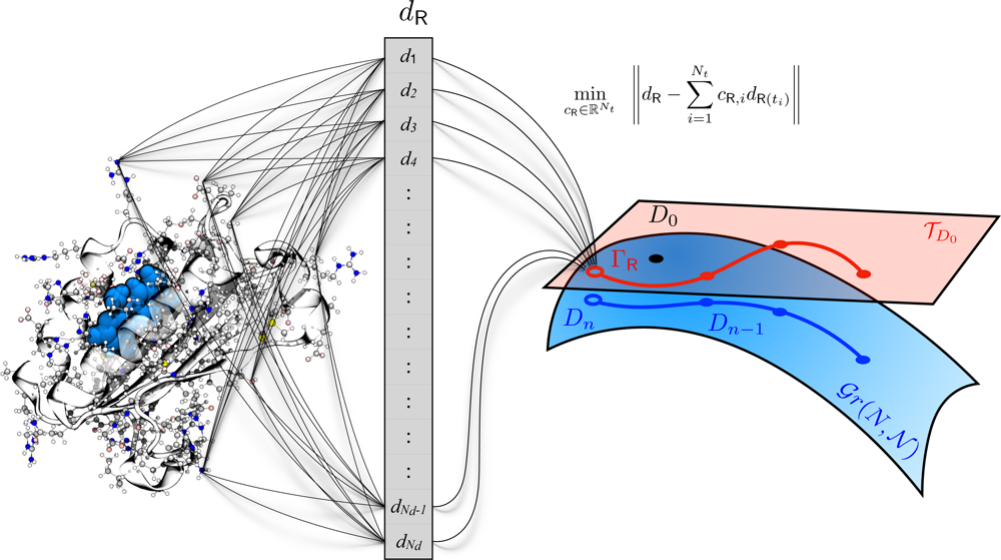

Born–Oppenheimer molecular dynamics (BOMD) is a powerful but expensive technique. The main bottleneck in a density functional theory BOMD calculation is the solution to the Kohn–Sham (KS) equations that requires an iterative procedure that starts from a guess for the density matrix. Converged densities from previous points in the trajectory can be used to extrapolate a new guess; however, the nonlinear constraint that an idempotent density needs to satisfy makes the direct use of standard linear extrapolation techniques not possible. In this contribution, we introduce a locally bijective map between the manifold where the density is defined and its tangent space so that linear extrapolation can be performed in a vector space while, at the same time, retaining the correct physical properties of the extrapolated density using molecular descriptors. We apply the method to real-life, multiscale, polarizable QM/MM BOMD simulations, showing that sizeable performance gains can be achieved, especially when a tighter convergence to the KS equations is required.

## 1 Introduction

Ab initio Born–Oppenheimer molecular dynamics (BOMD) is one of the most powerful and versatile techniques in computational chemistry, but its computational cost represents a big limitation to its routine use in quantum chemistry. To perform a BOMD simulation, one needs to solve the quantum mechanics (QM) equations, usually Kohn–Sham (KS) density functional theory (DFT), at each step, before computing the forces and propagating the trajectory of the nuclei. The iterative self-consistent field (SCF) procedure is expensive, as it requires to build, at each iteration, the KS matrix and to diagonalize it. Convergence can require tens of iterations, making the overall procedure, which has to be repeated a very large number of times, very expensive. To reduce the cost of BOMD simulations, it is therefore paramount to be able to perform as little iterations as possible while, at the same time, obtaining an SCF solution accurate enough to afford stable dynamics. From a conceptual point of view, at each step of a BOMD simulation, a map is built from the molecular geometry to the SCF density and then to the energy and forces. The former map, in practice, requires the solution to the SCF problem and is not only very complex but also highly nonlinear. However, the propagation of the molecular dynamics (MD) trajectory uses short, finite time steps so that the converged densities at previous steps, and thus at similar geometries, are available. As a consequence, the geometry to the density map can be in principle approximated by extrapolating the available densities at previous steps. The formulation of effective extrapolation schemes has been the object of several previous works.^1^ Among the proposed strategies, one for density matrix extrapolation was developed by Alfè,^2^ as a generalization of the wavefunction extrapolation method by Arias et al.,^3^ which is based on a least-squares regression on a few previous atomic positions. The main difficulty in performing an extrapolation of the density matrix stems from the nonlinearity of the problem. In other words, a linear combination of idempotent density matrices is not an idempotent density matrix, as density matrices are elements of a manifold and not of a vector space. To circumvent this problem, strategies that extrapolate the Fock or KS matrix^4,5^ or that use orbital transformation methods^6−8^ have been proposed.

A completely different strategy has been proposed by Niklasson and co-workers.^9−11^ In the extended Lagrangian Born-Oppenheimer (XLBO) method, an auxiliary density is propagated in a time-reversible fashion and then used as a guess for the SCF procedure. The strategy is particularly successful, as it combines an accurate guess with excellent stability properties. In particular, the XLBO method allows one to perform accurate simulations converging the SCF to average values [for instance, 10^–5^ in the root-mean-square (RMS) norm of the density increment], which are usually insufficient to compute accurate forces. An XLBO-based BOMD strategy has been recently developed by some of us in the context of polarizable multiscale BOMD simulations of both ground and excited states.^12−15^ Multiscale strategies can be efficiently combined, in a focused model spirit, to BOMD simulations to extend the size of treatable systems. Using a polarizable embedding allows one to achieve good accuracy in the description of environmental effects, especially if excited states or molecular properties are to be computed. In such a context, the XLBO guessing strategy allows one to perform stable simulation even using a modest 10^–5^ RMS convergence threshold, which, thanks to the quality of the XLBO guess, typically requires only about four SCF iterations. Recently, SCF-less formulations of the XLBO schemes have also been proposed.^16,17^

Unfortunately, the performances of the XLBO-based BOMD scheme are not so good when a tighter SCF convergence is required, which can be the case when one wants to perform MD simulations using post Hartree–Fock (HF) methods or for excited states described in a time-dependent DFT framework.^14,18^ In fact, such methods require the solution to a second set of QM equations which are typically nonvariational, making them more susceptible to numerical errors and instabilities. Computing the forces for non-SCF energies therefore requires a more accurate SCF solution.

The present work builds on all previous methods for density matrix extrapolation and aims at proposing a simple framework to overcome the difficulties associated with the nonlinearity of the problem. The strategy that we propose is based on a differential geometry approach and is particularly simple. First, we introduce a molecular descriptor, that is, a function of the molecular geometry and other molecular parameters that represent the molecular structure in a natural way that respects the invariance properties of the molecule within a vector space. At the (*n* + 1)-th step of an MD trajectory, we fit the new descriptor in a least-square fashion using the descriptors available at a number of previous steps and obtain a new set of coefficients. However, we do not use them to directly extrapolate the density. Instead, we first map the unknown density matrix that we aim to approximate from the manifold where it is defined to its tangent space. We then perform the extrapolation to approximate the representative density matrix in the tangent space, before mapping this approximation back to the manifold in order to obtain an extrapolated density matrix that satisfies the required physical constraints. This geometrical strategy, which has recently been introduced in the context of density matrix approximation by us,^19^ allows one to use standard linear extrapolation machinery without worrying about the nonlinear physical constraints on the density matrix, since both the space of descriptors and the tangent space are vector spaces. As the mapping between the manifold and the tangent space is locally bijective, no concerns about redundant degrees of freedom (such as rotations that mix occupied orbitals) arise. The map and its inverse, which are known as Grassmann logarithm and exponential, are easily computed and the implementation of the strategy is straightforward. We shall denote this approach as Grassmann extrapolation (G-Ext).

In this contribution, we choose a simple, yet effective molecular descriptor, and, for the extrapolation, a least square strategy. These are not the only choices. As our strategy allows one to use any linear extrapolation technique, which can be in turn coupled with any choice of molecular descriptor, more advanced strategies can be proposed, including machine learning. Our approach ensures that the extrapolated density, independent of how it is obtained, satisfies all the physical requirements of a density stemming from a single Slater determinant.

The paper is organized as follows. In the upcoming Section 2, we present all necessary theoretical foundations required for the development and implementation of the presented G-Ext approach. Section 3 then presents detailed numerical tests illustrating the performance of the extrapolation scheme, including realistic applications of BOMD within a QM/molecular mechanics (MM) context before we draw the conclusion in Section 4.

## 2 Theory

We consider ab initio BOMD simulations where the position vector  evolves in time according to classical mechanics as
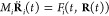
1where  denote the position of the *i*-th atom with mass *M*_*i*_, respectively, and the force acting on it at time *t*. We consider a general QM–MM method, but the setting also trivially applies to pure QM models. The forces at a given time *t* and position **R** of the nuclei arise from different interactions, namely, QM–QM, QM–MM, and MM–MM interactions. The computationally expensive part is to determine the state of the electronic structure, which is modeled here at the DFT level with a given basis set of dimension . Note that considering HF instead of DFT would not change much in the presentation of the method. It consists of computing the instantaneous nonlinear eigenvalue problem
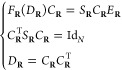
2where  and  denote the coefficients, respectively, of the occupied orbitals and density matrix and  denotes the diagonal matrix containing the energy levels. Furthermore,  denotes the DFT operator acting on the density matrix and  denotes the customary overlap matrix.

At this point, it is useful to note that the slightly modified coefficient matrix  belongs to the so-called Stiefel manifold defined as follows
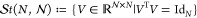
3due to the second equation in eq 2. In consequence, the normalized density matrix *D̃*_**R**_ = *C̃*_**R**_*C̃*_**R**_^T^ = *S*_**R**_^1/2^*D*_**R**_*S*_**R**_^1/2^ belongs to the following set
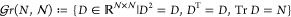
4which can be identified with the Grassmann manifold of *N*-dimensional subspaces of  by means of the spectral projectors. In the following, we always assume that the density matrix has been orthonormalized and therefore drop the ∼ from the notation. For any , one can associate the tangent space  which has the structure of a vector space. The evolution of the electronic structure can therefore be seen as a trajectory  on  where  denotes the trajectory of the nuclei.

The goal of the present work is to find a good approximation for the electronic density matrix at the next step of MD trajectory by extrapolating the densities at previous steps. More precisely, based on the knowledge of the density matrices , *i* = *n* – *N*_*t*_, ..., *n* – 1, at *N*_*t*_ previous times *t*_*i*_, one aims to compute an accurate guess of the density matrix *D*_*n*_ at time *t*_*n*_.

Thus, the problem formulation can be seen as an extrapolation problem of the following form: given the set of couples  and a new position vector  provide a guess for the solution *D*_*n*_. Here and in the remaining part of the article, we restrict ourselves on the positions of the QM atoms, that is, with slight abuse of notation, we denote from now on by **R** the set of QM positions only, even within a QM–MM context.

In order to approximate the mapping , we split this mapping in several submaps that will be composed as follows
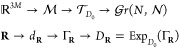
5where the first line shows the concatenation of maps in terms of spaces and the second in terms of variables. The different mappings will be presented and motivated in the following.

The first map is a mapping of the nuclear coordinates  to a (possibly high-dimensional) molecular descriptor that accounts for certain symmetries and invariances of the molecule. The last map, known as the Grassmann exponential, is introduced in order to obtain a resulting density matrix belonging to  and thus to guarantee that the guess fulfils all properties of a density matrix. As  is a manifold, this is not straightforward. The second mapping is the one that we aim to approximate but before we do that let us first introduce those two special mappings, that is, the molecular descriptor and the Grassmann exponential, in more details.

### 2.1 Molecular Descriptors

The map  is a map from atomic positions to molecular descriptors. These descriptors are used as fingerprints for the considered molecular configurations. Such molecular descriptors have been widely used in the past decades, for example, to learn potential energy surfaces (PESs)^20−26^ or to predict other quantities of interest. Among widely used descriptors, one can find Behler–Parinello symmetry functions,^27^ Coulomb matrix,^21^ smooth overlap of atomic positions (SOAP),^28^ permutationally invariant polynomials,^29^ or the atomic cluster expansion (ACE).^30,31^ These molecular descriptors are usually designed to retain similar symmetries as the targeted quantities of interest.

In this work, the quantity we are approximating is the density matrix, which is invariant with respect to translations and permutations of like particles. The transformation of the density matrix with respect to a global rotation of the system depends on the implementation, as it is possible to consider either a fixed Cartesian frame or one that moves with respect to the molecular system. In the former case, there is an equivariance with respect to rotations of the molecular system, while in the latter, the density matrix is invariant. We should therefore in principle use a molecular descriptor satisfying those properties.

However, the symmetry properties we will rely on are mostly translation and rotation invariance. Therefore, we will use a simple descriptor in the form of the Coulomb matrix denoted by *d*_**R**_, given by
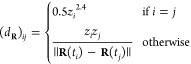
6

Note that such a descriptor is not invariant (nor equivariant) with respect to permutations of identical particles. However, we have found this descriptor to offer a good trade-off between simplicity and efficiency. Note that since we aim to extrapolate the density matrix from previous time steps, permutations of identical particles never occur from one time step to another and we do not need to rely on this property. Nevertheless, we expect that a better description could be achieved using more flexible descriptors, such as ACE polynomials or the SOAP descriptors, where the descriptors themselves can be tuned.

### 2.2 Grassmann Exponential

We only give a brief overview as the technical details have already been reported elsewhere,^19,32,33^ and are recapitulated in the Supporting Information. The set  is a smooth manifold and thus, at any point, say  in our application, there exists the tangent space  such that one can associate nearby points  to tangent vectors . The mapping  is known as the Grassmann logarithm and its inverse mapping is known as the Grassmann exponential . Furthermore,  and . These mappings are not only abstract tools from differential geometry but can also be computed by means of performing a singular value decomposition (SVD).^19,32,33^ In our application, we use the same reference point *D*_0_ in all cases which brings some computational advantages as will be discussed in more detail in the upcoming Section 2.3.

### 2.3 Approximation Problem

Since the tangent space  is a (linear) vector space, we can now aim to approximate the mapped density matrix on the tangent space . To simplify the presentation, we shift the indices in the following and describe the extrapolation method for the first *N*_*t*_ time steps. In the general setting, we should consider the positions **R**(*t*_*i*_) for *i* = *n* – *N*_*t*_, ..., *n* – 1, to extrapolate the density matrix at position **R**(*t*_*n*_), where *n* is the current time step of the MD. We look for parameter functions *c*_*i*_ such that, given previous snapshots Γ_*i*_ = Log(*D*_*i*_) for *i* from 1 to *N*_*t*_, corresponding to some **R**(*t*_*i*_)’s, the approximation of any density matrix on the tangent space is written as
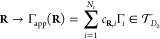
7with .

The question is then how to find these coefficient functions  and we propose to find those via the resolution of a (standard) least-square minimization problem. For a given position **R**, we look for coefficients that minimize the -error between the descriptor *d*_**R**_ and a linear combination of the previous ones *d*_**R**(*t*i)_
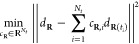
8

In the matrix form, this simply reads
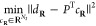
9where *P* is the matrix of size *N*_*t*_ × *N*_*d*_ containing the descriptors *P*_*i*,*j*_ ≔ (*d*_**R**(*t*_*i*_)_)_*j*_. Note that we only fit on the level of the descriptor, that is, the mapping from the position vector **R** to the descriptor *d*_**R**_ and that this method is similar to the ones used by Alfè^2^ and Arias et al.,^3^ where the descriptors they used were the positions of the atoms and only considered the previous three time steps of the MD.

If the system is underdetermined, we select the vector *c*_**R**_ that has the smallest norm. However, in general, the system is overdetermined as we have more descriptors than snapshots. This implies that this formulation verifies the interpolation principle: for every *i* and *j* from 1 to *N*_*t*_, the solution of problem (8) at the positions **R**(*t*_*j*_) satisfies *c*_**R**(*t*_*j*_)*i*_ = δ_*ji*_.

In principle, should we consider a large amount of previous descriptors, then the system may become undetermined and violate the interpolation principle. To mitigate this, we can use a stabilization scheme, as explained in the upcoming subsection.

Note that once we have computed the coefficients *c*_**R**_ by solving problem (12), one computes the initial guess for the density using the same coefficients in the linear combination on the tangent space as in eq 7 and finally takes the exponential (see eq 5). The rational for this step is that if the second mapping in eq 5, which we denote here by , was linear, then there would hold
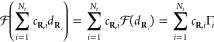
10

In practice, the mapping is, however, not linear and this approach works well in the test cases we considered. A possible explanation for this is the unfolding of the nuclear coordinates into a high-dimensional descriptor-space . Indeed, the high-dimensionality of  seems to allow an accurate approximation of  by a linear map. Furthermore, if the system is overdetermined, the scheme satisfies the interpolation property , and hence, we recover the expected density matrix .

#### 2.3.1 Stabilization

To stabilize the extrapolation by limiting high oscillations of the coefficients, we apply a Tikhonov regularization
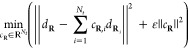
11for some choice of ε. This problem is always well-posed and corresponds to solving the following problem
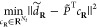
12where  is the vector *d*_**R**_ padded with *N*_*t*_ zeroes and  is the *P* matrix padded with the square diagonal matrix εId_*N*_*t*__. We observe in practice that using such a stabilization makes possible to use more previous points without degradation of the initial guess.

### 2.4 Final Algorithm

Given previous density matrices *D*_**R**(*t*_*j*_)_ for *j* = 1, ..., *N*_*t*_, the initial guess is computed following Algorithm 1. That is, we start by computing the logarithms of the density matrices *D*_**R**(*t*_*j*_)_, from the coefficients *C*_**R**(*t*_*j*_)_ that are first orthonormalized by performing *C̃*_**R**_ = *S*_**R**_^1/2^*C*_**R**_. Here, we remark that we assume that the density matrices *D*_**R**(*t*_*j*_)_ have been previously Löwdin orthonormalized.

We then compute the descriptors needed to build the *P̃* matrix and solve problem (12). This provides the coefficients in the linear combination of the Γ_*i*_’s on the tangent space. Finally, we compute the exponential of the linear combination in order to obtain the predicted density matrix.
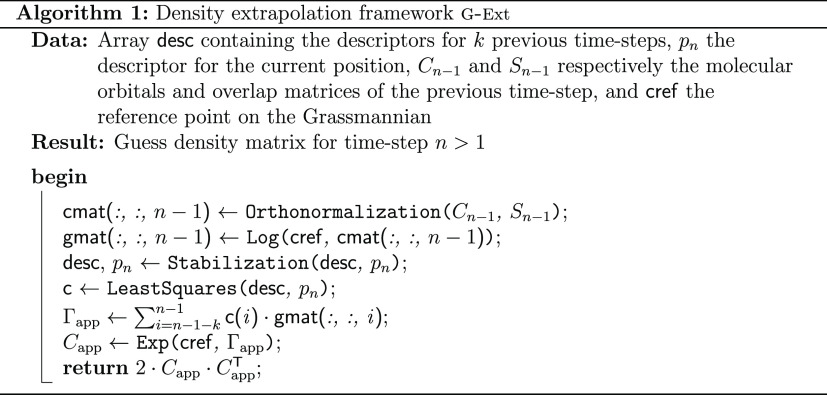


Note that the reference point *D*_0_ is chosen once and for all, which makes the computations of these logarithms lighter, even though there is no theoretical justification for keeping a single point *D*_0_ as a reference. Indeed, it is known that the formulae are only correct locally (around *D*_0_) on the manifold. However, in practice, we have never observed the need to change the reference point. This enables us to compute only one logarithmic map per time step and hence, only two SVDs in total per time step. To have a robust algorithm that will work even in this edge case, it will be sufficient to check that the exponential and logarithmic maps are still inverse of one another.

Finally, to be on the safe side with respect to the computations of the exponential, we have added a check on the orthogonality of the matrix that is obtained: if the residue is higher than a certain threshold, we then perform an orthogonalization of the result.

## 3 Numerical Tests

In this section, we present a series of numerical tests of the newly developed strategy. We test our method on four different systems. All the systems have been an object of a previous or current study by some of us and can therefore be considered representative of real-life applications.

The first system is 3-hydroxyflavone (3HF) in acetonitrile.^18^ Two systems (OCP and APPA) are chromophores embedded in a biological matrix—namely, a carotenoid in the orange carotenoid protein (OCP) and avine in acid phosphatase (APPA), a blue light-using flavine photoreceptor.^34−36^ The fourth system is dimethylaminobenzonitrile (DMABN) in methanol.^14^ The main characteristics of the systems used for testing are recapitulated in Table 1.

**Table 1:** Overview of the System Size in Terms of Number of QM Atoms (*N*_QM_), Number of MM Atoms (*N*_MM_), and the Total Number of QM Basis Functions

system	*N*_QM_	*N*_MM_	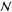
OCP	129	4915	1038
APPA	31	16,449	309
DMABN	21	6843	185
3HF	28	15,018	290

The systems used for testing include a quite large QM chromophore, the OCP, and three medium-sized systems, embedded in large- (APPA and 3HF) and medium-sized environments (DMABN), and are representative of different possible scenarios.

To test the performances of the new G-Ext strategy, we performed three sets of short (1 ps) multiscale BOMD simulations on OCP, APPA, 3HF, and DMABN. KS density functional theory was used to model the QM subsystem, using the B3LYP^37^ hybrid functional and Pople’s 6-31G(d) basis set.^38^ For the stability and energy conservation of the method, we did a longer and more realistic simulations of 10 ps on 3HF, where the flavone moiety was described using the ωB97X hybrid functional^39^ and Pople’s 6-31G(d) basis set. In all cases, the environment was modeled using the AMOEBA polarizable force field.^40^

All the simulations have been performed using the Gaussian–Tinker interface previously developed by some of us.^12,13^ In particular, we use a locally modified development version of Gaussian^41^ to compute the QM, electrostatic, and polarization energy and forces and Tinker^42^ to compute all other contributions to the QM/MM energy. We implemented the G-Ext extrapolation scheme in Tinker that acts as the main driver for the MD simulation, being responsible of summing together all the various contributions to the forces and propagating the trajectory. At each MD step, using the GauOpen interface,^43^ the density matrix, molecular orbital (MO) coefficients, and overlap matrix produced by Gaussian are retrieved. These are used to compute the extrapolated density, as described in Section 2. The density is then passed back to Gaussian to be used for the next MD step. All the simulations were carried out in the NVE ensemble, using the velocity Verlet integrator and a 0.5 fs time step. Concerning stabilization, we found that good overall results were obtained using a parameter ε ≔ 10^3^ × *r*_scf_, where *r*_scf_ is the tolerance of the SCF algorithm.

### 3.1 Numerical Results

To assess the performance of the G-Ext guess, we perform 1 ps MD simulations on the four systems described in Section 3 starting from the same exact conditions (positions and initial velocities) and using various strategies to compute the guess density for the SCF solver. We compare various flavors of the G-Ext method with the XLBO extrapolation scheme.^10^ Here, we note that the original XLBO method performs a propagation of an auxiliary density matrix, which is then used as a guess. The latter is not idempotent: to restore such a property, we perform a purification step at the beginning of the SCF procedure using McWeeny’s algorithm.^44^ In the following, we therefore compare our method, where we use 3 to 6 previous points for the fitting and extrapolation, to both the standard XLBO and to XLBO followed by purification (XLBO/MW). We use an SCF convergence threshold of 10^–5^ with respect to the RMS variation of the density.

We report in Table 2, for each method, the average number of SCF iterations performed along the MD simulation together with the associated standard deviation. As the XLBO strategy requires eight previous points, during which a standard SCF is performed, we discard the first points from the evaluation of the aforementioned quantities to have a fairer comparison.

**Table 2:** Performances of the G-Ext Method for Different Numbers of Extrapolation Points, Compared with the XLBO Algorithm with and without McWeeny Purification^a^

	OCP		DMABN		APPA		3HF	
method	average	σ	average	σ	average	σ	average	σ
XLBO	3.82	0.66	3.98	0.16	3.00	0.03	4.00	0.14
XLBO/MW	2.95	0.31	3.76	0.56	3.00	0.34	3.96	0.31
G-Ext(3)	2.57	0.84	3.54	0.78	2.95	0.50	3.09	0.41
G-Ext(4)	2.48	0.88	3.14	0.62	2.51	0.50	3.25	0.68
G-Ext(5)	2.25	0.96	3.23	0.75	2.51	0.50	3.30	0.72
G-Ext(6)	2.20	0.96	2.99	0.02	2.51	0.50	3.14	0.56

a All the results were obtained using a 10^–5^ convergence threshold for the RMS increment of the density matrix and are derived from a 1 ps long MD simulation, using a 0.5 fs time step. We report the average number of iterations required to converge the SCF, together with the associated standard deviation. Note that the first eight steps were discarded.

We do not report the total time required to compute the guess, as it is in all cases very small (up to 0.1 s wall clock time for the largest system using the G-Ext(6) guess). This is an important consideration, as the G-Ext method requires one to perform various linear-algebra operations (in particular, thin SVD) that can in principle be expensive. Thanks to the availability of optimized LAPACK libraries, this is in practice not a problem.

From the results in Table 2, we see that the G-Ext algorithms systematically outperforms the XLBO method. It is interesting to note that the McWeeny purification step has a sizeable effect on the performances of the XLBO method only for the largest system, OCP, where it results in the gain of almost one SCF iteration on average. On the other systems, the purification step has a smaller effect.

In all the systems we tested, the performances of the G-Ext method are systematically better than in XLBO, including McWeeny purification. The effectiveness of the G-Ext extrapolation increases when going from 3 to 6 points but quickly stagnates. We have performed further tests with more than 6 (up to 20) extrapolation points but never noted any further gain.

We observe a reduction in the number of iterations that goes from 0.5 in DMABN to 0.75 in OCP (1.62 when compared to XLBO without McWeeny purification). We remark that these gains, while apparently not so large, are greatly amplified during the MD simulation, due to the large number of steps that need to be performed.

The tests performed with a 10^–5^ convergence threshold are representative of a standard, DFT ground-state BOMD simulation. When performing a more sophisticated quantum mechanical calculation, such as a BOMD on an excited-state PES,^18^ such a convergence threshold may not be sufficient to guarantee the stability of the simulation, as the SCF solution is used to set up the linear response equations and the numerical error can be amplified, resulting in poorly accurate forces.

We tested the G-Ext algorithm in its best-performing version, the one that uses six extrapolation points, with a tighter, 10^–7^ threshold, again for the RMS variation of the density. The results are reported in Table 3, where we compare the G-Ext(6) scheme with the XLBO method with McWeeny purification.

**Table 3:** Performances of the G-Ext(6) Method Compared with the XLBO Algorithm with McWeeny Purification^a^

	OCP		DMABN		APPA		3HF	
method	average	σ	average	σ	average	σ	average	σ
XLBO/MW	5.02	0.17	7.30	0.64	7.49	0.84	7.47	0.63
G-Ext(6)	3.58	0.79	4.23	0.50	4.39	0.57	6.81	0.78

a All the results were obtained using a 10^–7^ convergence threshold for the RMS increment of the density matrix and are derived from a 1 ps long MD simulation, using a 0.5 fs time step. We report the average number of iterations required to converge the SCF, together with the associated standard deviation. Note that the first eight steps were discarded.

The XLBO method is based on the propagation of an auxiliary density, and therefore, the accuracy of the guess it generates depends little on the accuracy of the previous SCF densities. As a consequence, its performances are reduced if a tighter convergence is required. The G-Ext guess, on the other hand, uses previously computed densities as its building blocks and one can expect the accuracy of the resulting guess to be linked to the convergence threshold used during the simulation.

This is exactly what we observe. Using a threshold of 10^–7^, the G-Ext(6) guess exhibits significantly better performances than XLBO, gaining, on average, from about 0.7 to about 3 SCF iterations on the tested systems.

#### 3.1.1 Stability

The good performances of the G-Ext guess come, however, at a price, namely, the lack of time reversibility. We can thus expect the total energy in an NVE simulation to exhibit a long-time drift (LTD). Time reversibility and long-time energy conservation are, on the other hand, one of the biggest strengths of the XLBO method.

To investigate the stability of BOMD simulations using the G-Ext guess, we build a challenging case, where we start a BOMD simulation far from well-equilibrated conditions. We use the 3HF system as a test case and achieve the noisy starting conditions by starting from a well-equilibrated structure and changing the DFT functional from B3LYP to ωB97XD. This way, we have a physically acceptable structure, with no close atoms or other problematic structural situations, but obtain starting conditions that are far from equilibrium.

**Figure 1:**
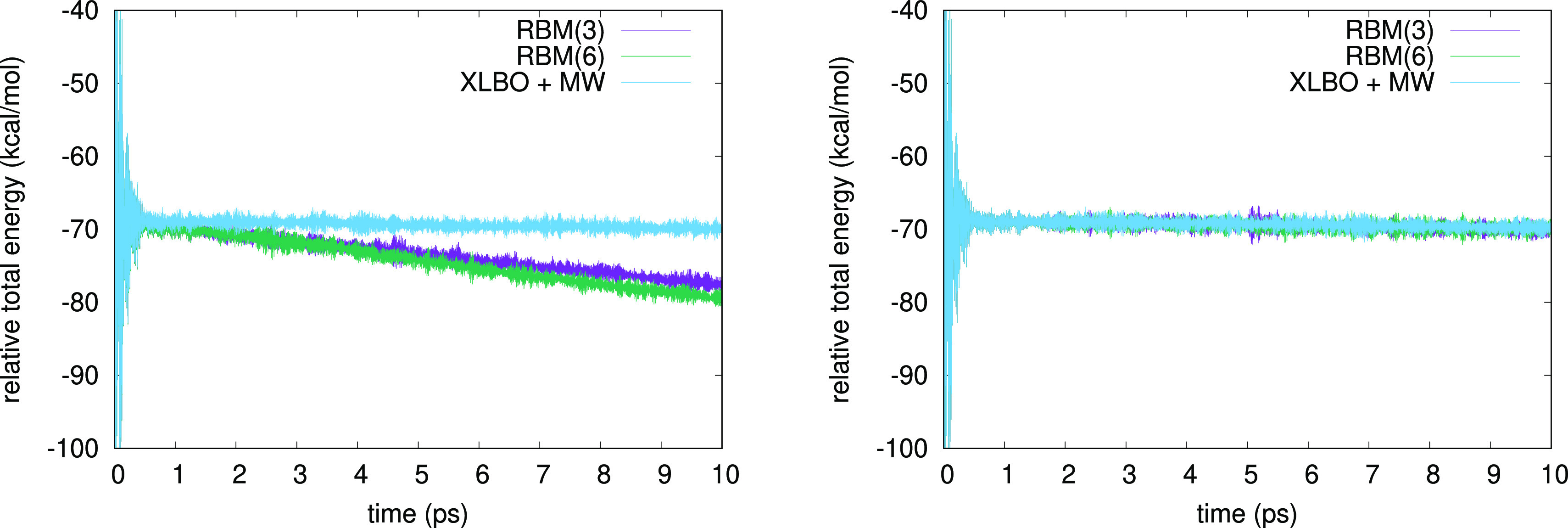
Total energy (kcal/mol) as a function of simulation time (fs) for 3HF comparing G-Ext(3), G-Ext(6), and XLBO with McWeeny purification, using a convergence threshold for the SCF algorithm of 10^–5^ (left panel) and 10^–7^ (right panel). The total energy was shifted to +505,000 kcal/mol for readability.

We report in Figure 1 the total energy along a 10 ps BOMD simulation of 3HF in acetonitrile using either a 10^–5^ SCF convergence threshold (left panel) or a 10^–7^ one (right panel). The same results for a 10^–6^ threshold are reported in the Supporting Information. We compare the G-Ext(3) and G-Ext(6) methods to the XLBO one including McWeeny purification. As already noted, while in principle the purification may spoil the time reversibility, this has no noticeable effect in practice.

The very noisy starting conditions are apparent from the energy profiles that exhibit large oscillations in the first couple of hundreds of femtoseconds.

To better estimate the short- and long-time energy stability, we report in Table 4 the average short-time fluctuation (STF) and LTD of the energy. The former is computed by taking the RMS of the energy fluctuation every 50 fs and averaging the results over the trajectory, discarding the first 500 fs, the latter by fitting the energy with a linear function and taking the slope.

**Table 4:** Short- and Long-Term Stability Analysis of the G-Ext(3) and G-Ext(6) Methods, Compared to the XLBO Algorithm with McWeeny Purification, for the 3HF System^a^

	conv. 10–^5^		conv. 10–^6^		conv. 10–^7^	
method	STF	LTD	STF	LTD	STF	LTD
XLBO/MW	0.55	–0.04	0.55	–0.03	0.57	–0.03
G-Ext(3)	0.55	–0.42	0.57	–0.15	0.53	–0.04
G-Ext(6)	0.56	–0.53	0.52	–0.13	0.57	–0.04

a For each method, we report the STF and the LTD and the average number of SCF iterations, for three convergence thresholds of the SCF algorithm.

All methods show comparable short-term stability, which is to be mainly ascribed to the chosen integration time step. On the other hand, from both the results in Table 4 and Figure 1, we observe a clear drift of the energy when the G-Ext method is used. In particular, the system cools of about 10 kcal/mol with either G-Ext(3) or G-Ext(6). The XLBO trajectory, despite the McWeeny purification, exhibits an almost perfect energy conservation.

These results are not surprising but should be taken into account when choosing to use the G-Ext guess, which, if coupled to a 10^–5^ SCF convergence threshold, cannot guarantee long-term energy conservation. The drift is overall not too large and can be handled using a thermostat. Whether or not the trade between performances and energy conservation is acceptable for a production simulation is a decision that ultimately lies with the user.

Increasing the accuracy of the SCF computation improves the overall stability for G-Ext, which is already good at 10^–6^ and becomes virtually identical to the one offered by the XLBO method at 10^–7^.

## 4 Conclusions

In this contribution, we presented an extrapolation scheme to predict initial guesses of the density matrix for the SCF iterations within BOMD. What makes our approach new is that we enforce the idempotency of the density matrix by extrapolating not the densities themselves but their map onto a vector space, which is the tangent plane to the manifold of the physically acceptable densities. Such a map is locally bijective so that after performing the extrapolation, we can map the new density back to the original manifold, providing thus an idempotent density. The main element of novelty of the algorithm is that by working on a tangent space, it allows one to use any linear extrapolation technique, while, at the same time, automatically ensuring the correct geometrical structure of the density matrix. As such, the technique presented in this paper can be seen as a simple case of a more general framework. Such a framework allows one to recast the problem of predicting a guess density by extrapolating information available from previous MD steps as a mapping between two vector spaces, that is, the space of molecular descriptors and the tangent plane. This geometric approach can be seen as an alternative to extrapolating quantities derived from the density, such as the Fock or Kohn–Sham matrix, as proposed by Pulay and Fogarasi^4^ and by Herbert and Head-Gordon.^5^ However, the framework we developed, using molecular descriptors and a general linear extrapolation technique, can in principle be easily extended to such approaches.

That being said, our choices of both the molecular descriptor and of the extrapolation strategies are far from being unique. In recent years, molecular descriptors gained attraction within the rise of machine-learning (ML) techniques. Our choice, namely, using the Coulomb matrix, is only one of the many possibilities, and while being simple and effective, more advanced descriptors may be used and possibly improve the overall performances of the method. We also used a straightforward (stabilized) least-square interpolation of the descriptors at the previous point to compute the extrapolation coefficients for the densities. This strategy is, again, simple yet effective. However, many other approaches can be used. In particular, ML techniques may not only provide a very accurate approximated map but also benefit of a larger amount of information (i.e., use the densities computed at a large number of previous steps), further improving the accuracy of the guess. Improvements on the descriptors and extrapolation strategies are not the only possible extensions of the proposed method. A natural extension that is under active investigation is the application to the G-Ext guess to geometry optimization, for which the XLBO scheme cannot be used.

Overall, even the simple choices made in this contribution produced an algorithm that exhibits promising performances. In all our tests, the G-Ext method outperformed the well-established XLBO technique, especially for tighter SCF accuracies which may be relevant for post-SCF BOMD computations, including computations on excited-state PES. While we tested the method only for KS DFT, it can also be used for HF or semiempirical calculations. The main disadvantage of the proposed strategy with respect to the XLBO method is, however, the lack of time reversibility, which manifests itself as a lack of long-term energy conservation. In particular, for longer MD simulations, the total energy may exhibit a visible drift, which is something that the user must be aware of. In our test, the observed drift was relatively small and the use of a thermostat should be enough to avoid problems in practical cases; however, this is a clear, and expected, limitation of the proposed approach. We note that using a tighter SCF convergence, which is also the case where the proposed method shows its best performances and produces an energy conserving trajectory, even starting from very noisy conditions. A time-reversible generalization of the G-Ext method is anyways particularly attractive and is at the moment under active investigation.

## Supporting Information Available

Julia template of the G-Ext algorithm is available at https://github.com/epolack/GExt.jl, figure representing the total energy computation with an SCF convergence threshold of 10^–6^ for the molecule 3HF, and formulae for the exponential and logarithm functions (PDF)
