# Supplementary material for: Grassmann Extrapolation of Density Matrices for Born–Oppenheimer Molecular Dynamics
Source: J Chem Theory Comput. 2021 Oct 8;17(11):6965–73. doi: 10.1021/acs.jctc.1c00751 (PMC8582259; doi:10.1021/acs.jctc.1c00751)
Supplement: Supplementary file 1 — ct1c00751_si_001.pdf [file ct1c00751_si_001.pdf]

Grassmann extrapolation of density matrices for Born-Oppenheimer molecular dynamics

Supplementary information

Étienne Polack Geneviève Dusson Benjamin Stamm
Filippo Lipparini

September 17, 2021

Supplementary figure

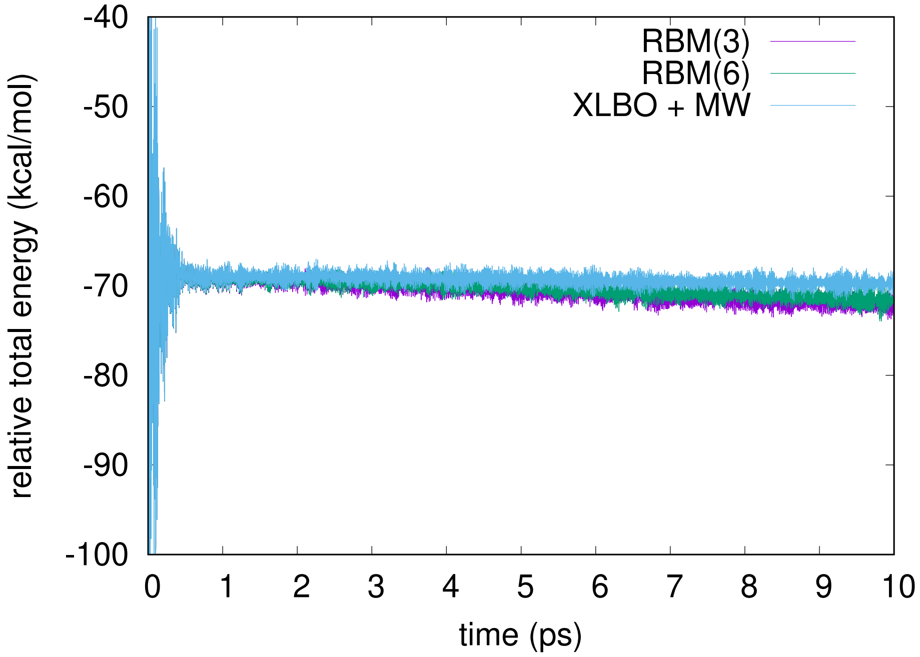

Figure 1: Total energy (kcal/mol) as a function of simulation time (fs) for 3HF comparing G-Ext(3), G-Ext(6) and XLBO with McWeeny purification, using a convergence threshold for the SCF algorithm of 10^{-6}. The total energy was shifted of $+505\,000$ kcal/mol for readability.

Grassmann Exponential and Logarithm maps

The Grassmann manifold is a differential manifold and, for any given $D_0 = C_0 C_0^\mathsf{T} \in \mathcal{G}r(N, \mathcal{N})$ with $D_0 := D_{\mathsf{R}_0}$ and $C_0 := C_{\mathsf{R}_0}$ for fixed R_0, the tangent space is characterized by

$$\mathcal{T}_{D_0} = \left\{ \Gamma \in \mathbb{R}^{\mathcal{N} \times N} \,\middle|\, C_0^\mathsf{T} \Gamma = 0 \right\} \subset \mathbb{R}^{\mathcal{N} \times N}. \tag{1}$$

Note that the tangent space is a linear space. One can then introduce the Grassmann exponential which maps tangent vectors on $\mathcal{T}_{D_0}$ to the manifold $\mathcal{G}r(N, \mathcal{N})$ in a locally bijective manner around D_0. Indeed, it is not only an abstract tool from differential geometry, but it can be computed in practice involving the matrix exponential. By complementing C_0 with orthonormal columns to obtain $(C_0, C_\perp) \in O(\mathcal{N})$, where $O(\mathcal{N})$ denotes the group of orthogonal matrices of dimension $\mathcal{N} \times \mathcal{N}$, and $\Gamma \in \mathcal{T}_{D_0}$ we have

$$\mathrm{Exp}_{D_0}(\Gamma) = C C^\mathsf{T}, \qquad C = \big(C_0, C_\perp\big) \exp \begin{pmatrix} 0 & -B^\mathsf{T} \\ B & 0 \end{pmatrix} \mathsf{I}_{\mathcal{N},N}. \tag{2}$$

Here, exp denotes the matrix exponential function, the matrix $B \in \mathbb{R}^{(\mathcal{N}-N) \times N}$ contains expansion coefficients of columns of Γ in a span of columns of $C_\perp$ such that $\Gamma = C_\perp B$ and $\mathsf{I}_{\mathcal{N},N} = (\mathsf{I}_N, 0)^\mathsf{T} \in \mathbb{R}^{\mathcal{N} \times N}$ are the first N columns of the $\mathcal{N} \times \mathcal{N}$ identity matrix. As described in [1, 2], the Grassmann exponential can then be equivalently expressed by

$$\mathrm{Exp}_{D_0}(\Gamma) = C C^\mathsf{T}, \qquad C = \left[C_0 V_e \cos(\Sigma_e) + U_e \sin(\Sigma_e)\right] V_e^\mathsf{T}, \tag{3}$$

by means of a singular value decomposition (SVD) of the matrix $\Gamma = U_e \Sigma_e V_e^\mathsf{T}$.

The inverse function is the so-called Grassmann logarithm Log_{D_0} (see, e.g., [1, 2]) which maps any $D = C C^\mathsf{T} \in \mathcal{G}r(N, \mathcal{N})$ in a neighborhood of D_0 to the tangent space $\mathcal{T}_{D_0}$ by

$$\mathrm{Log}_{D_0}(D) = U_\ell \arctan(\Sigma_\ell) V_\ell^\mathsf{T}, \tag{4}$$

using the following SVD decomposition

$$U_\ell \Sigma_\ell V_\ell^\mathsf{T} = L \qquad \text{with} \qquad L = C \left(C_0^\mathsf{T} C\right)^{-1} - C_0. \tag{5}$$

References

[1] Alan. Edelman, Tomás A. Arias, and Steven T. Smith. The Geometry of Algorithms with Orthogonality Constraints. *SIAM J. Matrix Anal. Appl.*, 20(2):303–353, 1998-01-01.

[2] Ralf Zimmermann. Manifold interpolation and model reduction, 2019. http://arxiv.org/abs/1902.06502.
